# Study on the influencing factors of primary dysmenorrhea in female college students: Systematic review and meta-analysis

**DOI:** 10.1097/MD.0000000000040906

**Published:** 2024-12-06

**Authors:** Jingyu Liu, Yimu Wang, Lingsha Wu, Lingyu Wang, Haiyan Fang

**Affiliations:** a Department of Nursing, The First Affiliated Hospital of Bengbu Medical University, Bengbu, China; b College of Nursing, Anhui University of Chinese Medicine, Hefei, China; c Department of Nursing, Jiaxing Second Hospital, Jiaxing, China.

**Keywords:** female college student, incidence rate, influencing factor, meta-analysis, primary dysmenorrhea

## Abstract

**Background:**

The influencing factors of primary dysmenorrhea in female college students were analyzed through meta-analysis to provide the corresponding basis for its prevention and treatment.

**Methods:**

The databases, including China National Knowledge Infrastructure, Wanfang Data Knowledge Service Platform, VIP database, China Biology Medicine Disc, Pubmed, Embase, Cochrane library, and Web of science were searched for the literature on the influencing factors of primary dysmenorrhea in female college students was retrieved from the science database from the establishment of the database to July 17, 2023. The Newcastle-Ottawa Quality Scale was used to score the quality of cohort and case–control studies included in the study. The cross-sectional studies were scored by the Agency for Healthcare Research and Quality. Two researchers independently screened the literature, and if there was no consensus, the third party would make a judgment on whether to include the literature. The extracted content included the first author, publication year, country, study type, sample size, and influencing factors. Stata17.0 software was used for meta-analysis.

**Results:**

A total of 23 studies were included, with a total sample size of 18,080 cases. Current evidence shows that the prevalence of primary dysmenorrhea in female college students is 70.3% (95%CI: 62.7–77.9%), and the combined odd ratio values (95%CI) of the main influencing factors are: family history of dysmenorrhea 2.116 (1.613–2.776), early age at menarche 2.200 (1.392–3.477), irregular menstrual cycle 1.662 (1.166–2.367), drinking cold drinks 1.717 (1.220–2.417), high caffeine intake 2.082 (1.379–3.144), stress 1.895 (1.515–2.282), medical specialty 1.827 (1.365–2.445), and adequate sleep 0.328 (0.232–0.463).

**Conclusion:**

The prevalence of primary dysmenorrhea is high in female college students, and adequate sleep is a protective factor for primary dysmenorrhea. Family history of dysmenorrhea, early age at menarche, irregular menstrual cycle, drinking cold drinks, high caffeine intake, stress, and medical specialty were all risk factors.

## 1. Introduction

Primary dysmenorrhea (PD) refers to periodic lower abdominal cramps that occur before or during menstruation in the female reproductive organs in the absence of other organic diseases.^[1]^ Female college students have a higher incidence of PD.^[2]^ PD can affect women’s sleep, mood, study and work during menstruation, and reduce the quality of life of patients to a certain extent.^[3]^ Therefore, in order to improve this situation, it is necessary to understand the influencing factors of PD in female college students. At present, some researchers have actively explored the current situation of PD in female college students, but due to differences in research design, sample size, and region, there are some differences in the reports on the influencing factors. This study intends to conduct a meta-analysis of the literature on PD in female college students in order to reveal the prevalence trend and influencing factors of PD in this population and provide corresponding evidence for the prevention and treatment of the disease.

## 2. Methods

### 2.1. Study registration

This study was registered in the international prospective register of systematic reviews (PROSPERO registration number CRD42023453313) and was conducted following the Preferred Reporting Items for Systematic Reviews and Meta-Analyses (PRISMA) statement. This article reports the results of a literature search and does not involve any animal, cell or human experimental research. This study did not require ethics approval in China.

### 2.2. Search strategy

**Figure 1.**
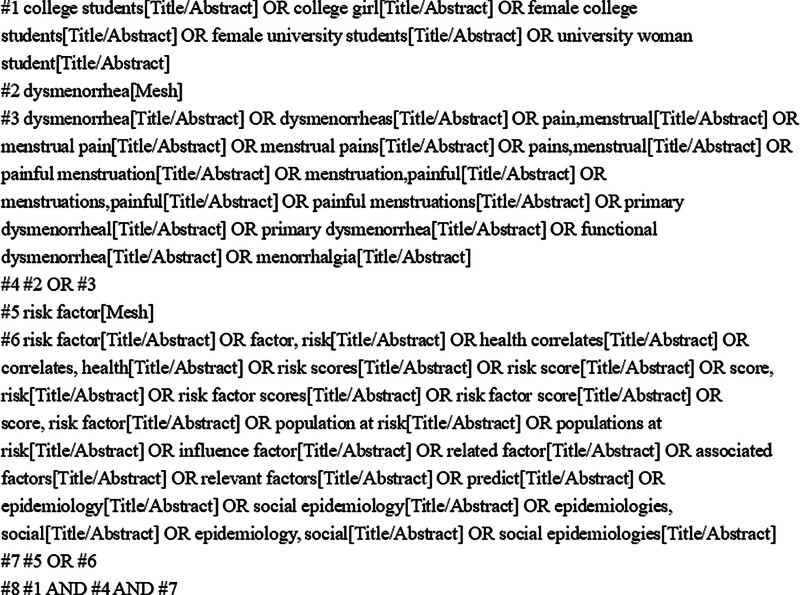
Retrieval strategy of Pubmed.

China National Knowledge Infrastructure, Wanfang Data Knowledge Service Platform, VIP database, China Biology Medicine Disc, Pubmed, Embase, Cochrane library and Web of science databases were searched by computer for Chinese and English literatures published from the establishment of the database to July 17, 2023. Search terms included: “female college students,” “primary dysmenorrhea,” “influencing factors,” “epidemiology,” etc. The literature search was completed by a combination of subject terms and free words, taking Pubmed as an example, and the specific search strategy is shown in Figure 1.

### 2.3. Eligibility and exclusion criteria

Inclusion criteria: (1) the type of study was cross-sectional, cohort or case–control study; (2) female college students; (3) clear diagnostic criteria for PD; (4) the odd ratio (OR) value and 95%CI were provided by original data OR obtained by data transformation. The exclusion criteria were: (1) systematic reviews, conferences, patents, reviews and other types of literature; (2) repeated publications (only 1 article with the most information retained); (3) literature with incomplete data; (4) full text of the literature was not available.

### 2.4. Risk of bias assessment

The included cross-sectional studies were evaluated using AHRQ evaluation criteria.^[4]^ The total score of the evaluation criteria was 0 to 11. The total score of literature was 0 to 3 as low quality, 4 to 7 as medium quality, and 8 to 11 as high quality. The included case–control studies and cohort studies were evaluated by the Newcastle-Ottawa Scale evaluation criteria,^[5]^ the total score of which was 0 to 9; a total score of ≥7 was considered to be of high quality. The quality evaluation of the literature was completed by 2 researchers independently. If there was any disagreement, the discussion was conducted, and the final result was decided by the third researcher.

### 2.5. Data extraction

Two researchers independently completed the literature screening in strict accordance with the inclusion and exclusion criteria. If there were any differences, they would discuss with each other and the third researcher would make a decision. After that, the data related content was extracted, and the method was the same as above. Data extraction included: first author, publication year, country, study type, sample size, and influencing factors.

### 2.6. Statistical analysis

Stata17.0 software was used to complete the corresponding statistical analysis. In this study, the combined effect value of PD prevalence in female college students was expressed by effect size (95%CI), and the combined effect value of influencing factors was expressed by OR (95%CI). The corresponding test was completed by Z test. If *P* < .05, the combined effect size was statistically significant. Heterogeneity between studies was assessed by I^2^ and Q tests. If I^2^ < 50% and *P* > .05, it was considered that there was homogeneity among multiple studies, and the fixed effect model was used. Otherwise, a random effects model was used. Sensitivity analysis was performed by comparing the consistency of the results between the fixed effect model and the random effect model. If the results were inconsistent, the studies that had a great impact on the results should be excluded for further analysis.

## 3. Results

### 3.1. Study selection

**Figure 2.**
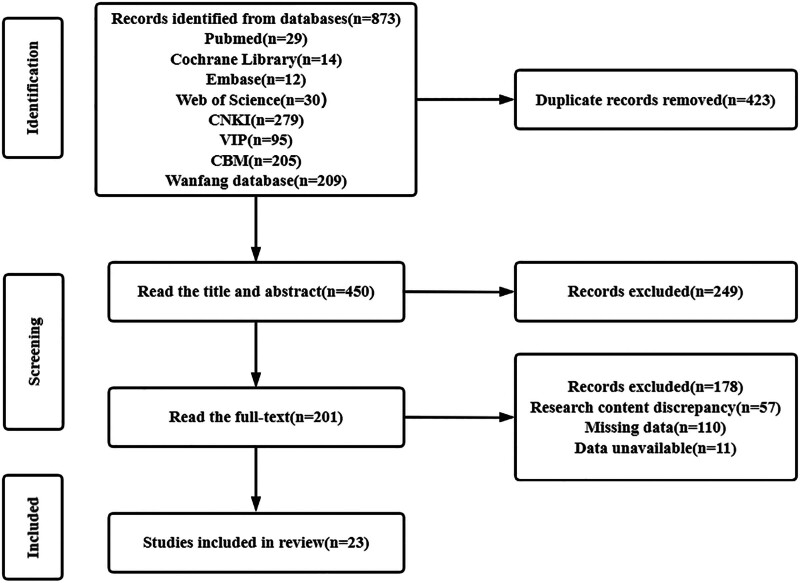
Literature screening flow chart.

A total of 873 literatures were retrieved, and 450 literatures were obtained after eliminating duplicate literatures. According to the inclusion criteria and exclusion criteria, 23 literatures^[6–27]^ were finally included. The literature screening process and results are shown in Figure 2.

### 3.2. Basic characteristics and quality evaluation of the included literature

Twenty cross-sectional studies^[6–8,10–15,17,19–27]^and 3 case–control studies^[9,16,18]^ were included in the meta-analysis. A total of 9 countries were involved, and the main research area was China. The quality of the literature was medium or high. As shown in Table 1.

**Table 1:** Basic information and quality evaluation of the included literature.

Author	Year	Region	Study type	Sample size	Number of patients	Prevalence rate	Influence factor	Quality score
Meng WL^[6]^	2013	China	Cross-sectional study	579	404	69.78	1, 2	6
Chen LJ^[7]^	2014	China	Cross-sectional study	1 574	1 023	65.00	2, 3, 4	8
Zhang N^[8]^	2016	China	Cross-sectional study	800	484	60.50	1	7
Yang Q^[9]^	2016	China	Case–control study	650	296	–	–	7
Bai JY^[10]^	2016	China	Cross-sectional study	1 154	860	74.50	5, 2	7
Liang XD^[11]^	2017	China	Cross-sectional study	583	453	77.70	2, 3	8
Wang HR^[12]^	2019	China	Cross-sectional study	1 069	768	71.80		8
Lu JY^[2]^	2021	China	Cross-sectional study	319	246	77.10	6, 2	7
Tao XX^[13]^	2022	China	Cross-sectional study	333	269	80.80	2, 7	7
Luo J^[14]^	2023	China	Cross-sectional study	1331	1013	76.11	8, 2	8
Zukri SM^[15]^	2009	Malaysia	Cross-sectional study	271	138	50.90	2	8
Faramarzi M^[16]^	2014	Iran	Case–control study	360	180	–	2, 9	7
Tomás-Rodríguez MI^[17]^	2017	Spain	Cross-sectional study	306	281	91.80	–	8
Bavil DA^[18]^	2018	Iran	Case–control study	250	125	–	5, 7	7
Fernández-Martínez E^[19]^	2018	Spain	Cross-sectional study	258	193	74.80	2	8
Zurawiecka M^[20]^	2018	Poland	Cross-sectional study	771	500	64.85	10, 8	8
Giletew A^[21]^	2019	Ethiopia	Cross-sectional study	183	114	62.30	8, 2, 11	9
Hu Z^[22]^	2020	China	Cross-sectional study	4 606	1 921	41.70	10, 2, 8, 11	9
Hashim RT^[23]^	2020	Saudi Arabia	Cross-sectional study	336	269	80.10	9	8
Ullah A^[24]^	2021	Pakistan	Cross-sectional study	600	549	91.5	8, 6, 11, 4	8
Tadese M^[25]^	2021	Ethiopia	Cross-sectional study	647	317	51.50	11, 2	9
Karout S^[26]^	2021	Lebanon	Cross-sectional study	550	445	80.90	6, 2	9
Mesele TT^[27]^	2022	Ethiopia	Cross-sectional study	550	356	64.7	8	8

*Note*: 1 = drink cold drinks; 2 = family history of dysmenorrhea; 3 = adequate sleep; 4 = stress; 5 = age; 6 = medical specialty; 7 = physical exercise; 8 = early age at menarche; 9 = high caffeine intake; 10 = underweight; 11 = irregular menstrual cycle.

### 3.3. Results of meta-analysis of PD prevalence

#### 3.3.1. Overall prevalence

**Figure 3.**
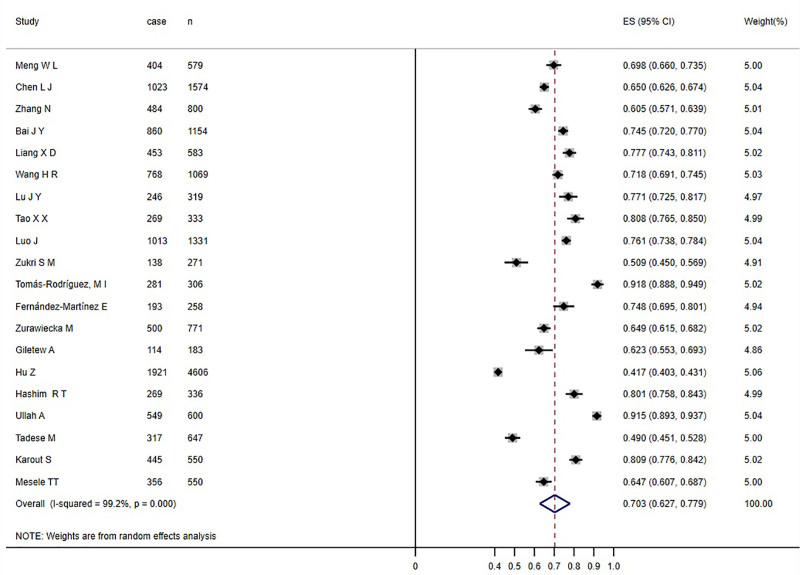
Forest map of PD among female college students. PD = primary dysmenorrhea.

Twenty studies reported the prevalence of PD in female college students, heterogeneity test I^2^ was 99.1% (*P* < .05), suggesting that there was a large heterogeneity, so the random effect model was used for analysis. Meta-analysis showed that the prevalence of PD in female college students was 70.3% (95%CI: 62.7–77.9%) (see Fig. 3).

#### 3.3.2. Results of subgroup analysis of PD prevalence

Subgroup analysis by region showed high heterogeneity between subgroups, and the random effects model was used for analysis. The results showed that the prevalence of PD in female college students in China (69.5%) was lower than that in other countries (71.2%), and the difference was statistically significant (*P* < .001), as shown in Table 2.

**Table 2:** Subgroup analysis of the prevalence of PD.

Analysis item	Number of studies	Sample size	Number of patients	Heterogeneity test		Results of meta-analysis		
				I^*2*^	*P*	Prevalence rate	95%CI	*P*
Region								<.001
China	10	16 307	10 083	99.3	<.001	69.5	(59.3, 79.6)	
Other countries	10	2 125	1 495	98.6	<.001	71.2	(61.2, 81.1)	

PD = primary dysmenorrhea.

### 3.4. Meta-analysis results of influencing factors of PD in female college students

A total of 11 influencing factors were extracted from the 23 included literatures for meta-analysis. The results showed that adequate sleep was a protective factor for PD in female college students, while family history of dysmenorrhea, early age of menarche, irregular menstrual cycle, high caffeine intake, stress, drinking cold drinks, and medical specialty were risk factors for PD. Underweight, physical exercise and age were not associated with PD (*P* > .05). As shown in Table 3.

**Table 3:** Meta-analysis of risk factors for PD.

Influence factor	Effect size		Heterogeneity test		Model of effect	Publication bias	
	OR (95%CI)	*P*	I^2^ (%)	*P*		Egger test (*P*)	Begg test (*P*)
Underweight^[20,22]^	1.916 (0.751, 4.936)	.172	88.7	.003	Random	–	1.000
Family history of dysmenorrhea^[2,6,7,10,11,13–16,19,21,22,25,26]^	2.116 (1.613, 2.776)	<.001	89.0	<.001	Random	.976	.743
Early age at menarche^[21,22,24,25]^	2.200 (1.392, 3.477)	.001	85.4	<.001	Random	.005	.009
Irregular menstrual cycle^[21,22,24,25]^	1.662 (1.166, 2.367)	.005	74.0	.009	Random	.099	.308
Physical exercise^[13,18]^	0.362 (0.035, 3.720)	.392	85.3	.009	Random	–	1.000
Age^[10,18]^	1.014 (0.721, 1.426)	.938	91.1	.001	Random	–	1.000
High caffeine intake^[16,23]^	2.082 (1.379, 3.144)	<.001	0.0	.801	Fixed	–	1.000
Adequate sleep^[7,11]^	0.328 (0.232, 0.463)	<.001	0.0	.677	Fixed	–	1.000
Stress^[7,24]^	1.895 (1.515, 2.282)	<.001	0.0	.969	Fixed	–	1.000
Drink cold drinks^[6,8]^	1.717 (1.220, 2.417)	.002	19.6	.265	Fixed	–	1.000
Medical specialty^[2,24,26]^	1.827 (1.365, 2.445)	<.001	0.0	.777	Fixed	.486	1.000

PD = primary dysmenorrhea.

### 3.5. Publication bias

Egger test and Begg test indicated that all the influencing factors were *P* > .05, indicating that there was little possibility of publication bias among the studies. As shown in Table 3.

### 3.6. Sensitivity analysis

Sensitivity analysis by random effect model and fixed effect model showed that the influencing factors were consistent and the results were stable. As shown in Table 4.

**Table 4:** Sensitivity analysis of risk factors for PD.

Influence factor	Random effect model	Fixed effect model
Underweight	1.916 (0.751, 4.936)	1.305 (1.143, 1.491)
Family history of dysmenorrhea	2.116 (1.613, 2.776)	2.077 (1.922, 2.245)
Early age at menarche	2.200 (1.392, 3.477)	1.355 (1.204, 1.524)
Irregular menstrual cycle	1.662 (1.166, 2.367)	1.338 (1.187, 1.507)
Physical exercise	0.362 (0.035, 3.720)	1.008 (1.000, 1.016)
Age	1.014 (0.721, 1.426)	1.000 (0.903, 1.106)
High caffeine intake	2.082 (1.379, 3.144)	2.082 (1.379, 3.144)
Adequate sleep	0.328 (0.232, 0.463)	0.328 (0.232, 0.463)
Stress	1.895 (1.515, 2.282)	1.895 (1.515, 2.282)
Drink cold drinks	1.756 (1.173, 2.628)	1.717 (1.220, 2.417)
Medical specialty	1.827 (1.365, 2.445)	1.827 (1.365, 2.445)

PD = primary dysmenorrhea.

## 4. Discussion

### 4.1. Prevalence of PD in female college students

The results of this study showed that the prevalence of PD in female college students was 70.3%, which was significantly higher than that reported by Li Dongmei^[28]^ (54.75%), but lower than that reported by Wu Jingjing^[29]^ (78.5%). It can be seen that the results of different studies are quite different, which may be caused by different study areas, sample size, etc. In this study, the female college students have a higher prevalence of PD, and corresponding prevention and treatment measures should be taken for this population as early as possible. Subgroup analysis showed that the prevalence of PD in China was lower than that in other countries, which might be related to the different constitution, living environment and living habits of female college students between the east and the west.

### 4.2. The effect of sleep on PD in female college students

The results of this study suggest that adequate sleep is a protective factor for PD. When sleep is sufficient, the secretion of follicle-stimulating hormone and luteinizing hormone increases, which can effectively maintain endocrine balance and avoid dysmenorrhea.^[7,11]^ If there is lack of sleep or poor sleep quality, hormone secretion will be reduced, prone to menstrual disorders, and long-term lack of sleep will appear anxiety, depression and other adverse emotions, easy to fatigue, seriously affect the body function, easy to progress to dysmenorrhea. Female college students should pay attention to their own good work and rest regularity, and arrange work and rest time reasonably according to the actual situation. Be fully aware of the many health risks that lack of sleep can cause, and actively avoid the erosion of normal sleep time by electronic information products and other recreational activities. We should ensure adequate sleep to reduce the risk of PD.

### 4.3. Influence of family history of dysmenorrhea, age at menarche and menstrual cycle on PD in female college students

A family history of PD is a risk factor for the onset of PD, which is consistent with the results of Habibi N et al.^[30]^ As an important influencing factor of PD, genetic factors may be the specific genes in the mother’s chromosome that make people’s temperament unstable, which transmit the relevant information to her daughter, making her susceptible to stimulation or pain threshold decreased and dysmenorrhea. It is also possible that the loss of GSTT1 gene increases the risk of dysmenorrhea.^[31,32]^ Early age at menarche and irregular menstrual cycle are risk factors for dysmenorrhea, but the underlying pathological mechanism is still unclear. A reasonable biological explanation is that women with early age at menarche are more likely to have irregular and prolonged menstruation after menarche, and excessive prostaglandin secretion during menstruation leads to uterine contraction and pain.^[33]^ A Palestinian study^[34]^ showed that students with irregular menstrual cycles were approximately twice as likely to experience dysmenorrhea. Liu Encheng et al^[35]^ mentioned that age is directly proportional to the physical and psychological maturity of patients. Early age of menarche, incomplete development of the body, coupled with the lack of corresponding awareness of menstrual health care, easy exposure to the predisposing factors of PD will also increase the risk of PD.

### 4.4. Effects of caffeine and cold drink intake on PD in female college students

Excessive caffeine intake is a risk factor for PD, which is consistent with the results of Al-MatouqS^[36]^ et al. However, there is still a lack of research on its exact mechanism of action. One possible explanation is that caffeine, the main component of coffee, is an adenosine analog that inhibits receptors for adenosine, a powerful vasodilator.^[37]^ Blocking these receptors causes blood vessels to constrict, which reduces blood flow to the uterus, further increasing the severity of period pain.^[38]^ Another possible explanation is that adenosine, as an important sleep regulator, may affect the circadian clock and the interaction between the circadian clock and the sleep homeostasis mechanism. Therefore, caffeine may affect sleep by changing the circadian clock function.^[39]^ Insufficient sleep is related to the occurrence of dysmenorrhea, and it may act on dysmenorrhea through this indirect effect. In this regard, we should try to avoid consuming caffeine in the evening, which will help treat the delayed sleep time problem through the circadian rhythm and established wake–sleep mechanism,^[40]^ thereby reducing the possibility of sleep quality affecting dysmenorrhea. However, it is important to note that the effects of the adenosine system and “adenosine” interventions on sleep and circadian systems observed in a series of caffeine studies may adapt over time and even show different or opposite effects than expected.^[41]^ For example, long-term caffeine intake can lead to reduced activity during the resting phase or increased non-rapid eye movement sleep.^[42,43]^ However, the time course of this adaptation (and its reversibility) is unclear. It may depend on dose, individual differences in metabolic processes, and differences in the adenosine system itself. In addition, a study comparing the intake of decaffeinated and caffeinated coffee found that the key factor affecting metabolic syndrome is not caffeine, but chlorogenic acid, another component of coffee.^[44]^ This may suggest that we should not limit ourselves to its main ingredient, caffeine, when exploring the mechanism of action of coffee on dysmenorrhea.

Cold drink intake is a risk factor for PD. It should be noted that both studies that mentioned cold drink intake were from China, and no studies on the influencing factor of cold drink intake have been found in other countries and regions. This situation may be related to regional cultural differences and racial differences. For example, people in Europe and the United States are more accustomed to drinking cold or ice water, but in China, people are more accustomed to drinking hot or warm water. Women in Europe and the United States can bathe in the cold water of the swimming pool immediately after giving birth, but in China, women need to recuperate in a warm indoor environment for 1 month or even longer after giving birth. During this period, it is strictly forbidden to eat raw or cold food or stay in a cold environment. In China, cold drinks refer to substances that can be consumed by humans, including iced or cold water and beverages. The adverse effects of cold drinks on dysmenorrhea are mainly the result of epidemiological surveys and studies. Currently, no research has provided a reasonable and scientific explanation of its mechanism of action from the perspective of pathophysiology. In traditional Chinese medicine theory, dysmenorrhea can be caused by “cold,” and Chinese medicine often uses methods such as moxibustion to expel cold and treat dysmenorrhea.^[45,46]^ Drinking cold drinks will increase the “cold” in the body, thereby aggravating the severity of dysmenorrhea. The mechanism by which cold drink intake causes dysmenorrhea needs to be studied in a more scientific and powerful way, and at least China should make more efforts to this end. In view of this, we should be cautious about the conclusion that cold drink intake is a risk factor for dysmenorrhea.

### 4.5. Influence of stress and medical major on PD of female college students

Stress and medical specialty as risk factors for PD indicate that there is a correlation between the occurrence of PD and psychosocial factors. Stress is generally considered to be a cognitive and behavioral experience process composed of psychological stress sources and psychological stress responses. The stress in this study mainly refers to learning stress. Stress has been shown to downregulate benzodiazepine receptors and activate hypothalamic–pituitary–adrenal axis responses, leading to the occurrence of pain.^[47]^ In addition, it has been shown that the magnitude of stress is a strong predictor of PD severity, and the magnitude of stress is positively correlated with the degree of pain in PD.^[24]^ Compared with other majors, medical students have heavier learning tasks and higher mental stress. Therefore, it is of great significance to learn to relieve stress and maintain a good psychological state for the prevention and remission of PD. However, since neither of the 2 studies on stress included in this study used professional scales to assess stress, we need to be cautious about the conclusions of this study.

### 4.6. Study limitations

This study also has some limitations. First, the heterogeneity among studies is high due to the differences in study areas. In addition, the number of articles covered by some influencing factors (high caffeine intake) was small, and the results of statistical analysis should be interpreted with caution.

## 5. Conclusions

In conclusion, the prevalence of PD is high in female college students. Adequate sleep is a protective factor for PD, while family history of dysmenorrhea, early age of menarche, irregular menstrual cycle, drinking cold drinks, high caffeine intake, stress, and medical specialty are risk factors for PD. It is of great significance to carry out targeted health education for female college students, especially for PD patients, to publicize the knowledge of PD and inform them of the related protection and risk factors, so as to reduce the incidence of PD and relieve pain.

## Author contributions

**Conceptualization:** Jingyu Liu, Lingsha Wu.

**Data curation:** Jingyu Liu, Yimu Wang.

**Formal analysis:** Jingyu Liu.

**Investigation:** Jingyu Liu.

**Methodology:** Jingyu Liu, Yimu Wang.

**Software:** Yimu Wang.

**Writing – original draft:** Jingyu Liu, Yimu Wang.

**Writing – review & editing:** Lingyu Wang, Haiyan Fang.
